# Comparing different supervised machine learning algorithms for disease prediction

**DOI:** 10.1186/s12911-019-1004-8

**Published:** 2019-12-21

**Authors:** Shahadat Uddin, Arif Khan, Md Ekramul Hossain, Mohammad Ali Moni

**Affiliations:** 1 Complex Systems Research Group, Faculty of Engineering, The University of Sydney, Room 524, SIT Building (J12), Darlington, NSW 2008 Australia; 2 Health Market Quality Research Stream, Capital Markets CRC, Level 3, 55 Harrington Street, Sydney, NSW Australia; 3 Faculty of Medicine and Health, School of Medical Sciences, The University of Sydney, Camperdown, NSW 2006 Australia

**Keywords:** Machine learning, Supervised machine learning algorithm, Medical data, Disease prediction

## Abstract

**Background:**

Supervised machine learning algorithms have been a dominant method in the data mining field. Disease prediction using health data has recently shown a potential application area for these methods. This study ai7ms to identify the key trends among different types of supervised machine learning algorithms, and their performance and usage for disease risk prediction.

**Methods:**

In this study, extensive research efforts were made to identify those studies that applied more than one supervised machine learning algorithm on single disease prediction. Two databases (i.e., Scopus and PubMed) were searched for different types of search items. Thus, we selected 48 articles in total for the comparison among variants supervised machine learning algorithms for disease prediction.

**Results:**

We found that the Support Vector Machine (SVM) algorithm is applied most frequently (in 29 studies) followed by the Naïve Bayes algorithm (in 23 studies). However, the Random Forest (RF) algorithm showed superior accuracy comparatively. Of the 17 studies where it was applied, RF showed the highest accuracy in 9 of them, i.e., 53%. This was followed by SVM which topped in 41% of the studies it was considered.

**Conclusion:**

This study provides a wide overview of the relative performance of different variants of supervised machine learning algorithms for disease prediction. This important information of relative performance can be used to aid researchers in the selection of an appropriate supervised machine learning algorithm for their studies.

## Background

Machine learning algorithms employ a variety of statistical, probabilistic and optimisation methods to learn from past experience and detect useful patterns from large, unstructured and complex datasets [1]. These algorithms have a wide range of applications, including automated text categorisation [2], network intrusion detection [3], junk e-mail filtering [4], detection of credit card fraud [5], customer purchase behaviour detection [6], optimising manufacturing process [7] and disease modelling [8]. Most of these applications have been implemented using supervised variants [4, 5, 8] of the machine learning algorithms rather than unsupervised ones. In the supervised variant, a prediction model is developed by learning a dataset where the label is known and accordingly the outcome of unlabelled examples can be predicted [9].

The scope of this research is primarily on the performance analysis of disease prediction approaches using different variants of supervised machine learning algorithms. Disease prediction and in a broader context, medical informatics, have recently gained significant attention from the data science research community in recent years. This is primarily due to the wide adaptation of computer-based technology into the health sector in different forms (e.g., electronic health records and administrative data) and subsequent availability of large health databases for researchers. These electronic data are being utilised in a wide range of healthcare research areas such as the analysis of healthcare utilisation [10], measuring performance of a hospital care network [11], exploring patterns and cost of care [12], developing disease risk prediction model [13, 14], chronic disease surveillance [15], and comparing disease prevalence and drug outcomes [16]. Our research focuses on the disease risk prediction models involving machine learning algorithms (e.g., support vector machine, logistic regression and artificial neural network), specifically - supervised learning algorithms. Models based on these algorithms use labelled training data of patients for training [8, 17, 18]. For the test set, patients are classified into several groups such as low risk and high risk.

Given the growing applicability and effectiveness of supervised machine learning algorithms on predictive disease modelling, the breadth of research still seems progressing. Specifically, we found little research that makes a comprehensive review of published articles employing different supervised learning algorithms for disease prediction. Therefore, this research aims to identify key trends among different types of supervised machine learning algorithms, their performance accuracies and the types of diseases being studied. In addition, the advantages and limitations of different supervised machine learning algorithms are summarised. The results of this study will help the scholars to better understand current trends and hotspots of disease prediction models using supervised machine learning algorithms and formulate their research goals accordingly.

In making comparisons among different supervised machine learning algorithms, this study reviewed, by following the PRISMA guidelines [19], existing studies from the literature that used such algorithms for disease prediction. More specifically, this article considered only those studies that used more than one supervised machine learning algorithm for a single disease prediction in the same research setting. This made the principal contribution of this study (i.e., comparison among different supervised machine learning algorithms) more accurate and comprehensive since the comparison of the performance of a single algorithm across different study settings can be biased and generate erroneous results [20].

Traditionally, standard statistical methods and doctor’s intuition, knowledge and experience had been used for prognosis and disease risk prediction. This practice often leads to unwanted biases, errors and high expenses, and negatively affects the quality of service provided to patients [21]. With the increasing availability of electronic health data, more robust and advanced computational approaches such as machine learning have become more practical to apply and explore in disease prediction area. In the literature, most of the related studies utilised one or more machine learning algorithms for a particular disease prediction. For this reason, the performance comparison of different supervised machine learning algorithms for disease prediction is the primary focus of this study.

In the following sections, we discuss different variants of supervised machine learning algorithm, followed by presenting the methods of this study. In the subsequent sections, we present the results and discussion of the study.

## Methods

### Supervised machine learning algorithm

At its most basic sense, machine learning uses programmed algorithms that learn and optimise their operations by analysing input data to make predictions within an acceptable range. With the feeding of new data, these algorithms tend to make more accurate predictions. Although there are some variations of how to group machine learning algorithms they can be divided into three broad categories according to their purposes and the way the underlying machine is being taught. These three categories are: supervised, unsupervised and semi-supervised.

**Fig. 1:**
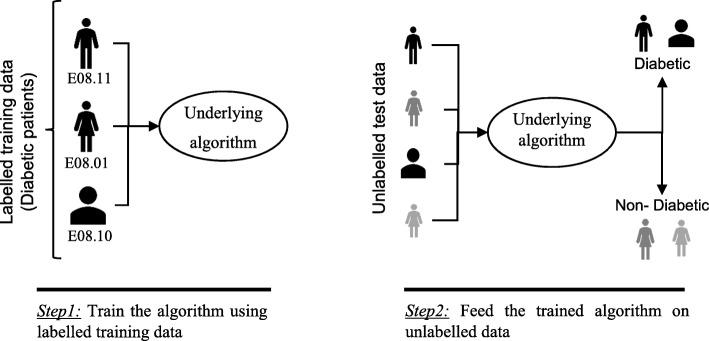
An illustration of how supervised machine learning algorithms work to categorise diabetic and non-diabetic patients based on abstract data

In supervised machine learning algorithms, a labelled training dataset is used first to train the underlying algorithm. This trained algorithm is then fed on the unlabelled test dataset to categorise them into similar groups. Using an abstract dataset for three diabetic patients, Fig. 1 shows an illustration about how supervised machine learning algorithms work to categorise diabetic and non-diabetic patients. Supervised learning algorithms suit well with two types of problems: classification problems; and regression problems. In classification problems, the underlying output variable is discrete. This variable is categorised into different groups or categories, such as ‘red’ or ‘black’, or it could be ‘diabetic’ and ‘non-diabetic’. The corresponding output variable is a real value in regression problems, such as the risk of developing cardiovascular disease for an individual. In the following subsections, we briefly describe the commonly used supervised machine learning algorithms for disease prediction.

#### Logistic regression

Logistic regression (LR) is a powerful and well-established method for supervised classification [22]. It can be considered as an extension of ordinary regression and can model only a dichotomous variable which usually represents the occurrence or non-occurrence of an event. LR helps in finding the probability that a new instance belongs to a certain class. Since it is a probability, the outcome lies between 0 and 1. Therefore, to use the LR as a binary classifier, a threshold needs to be assigned to differentiate two classes. For example, a probability value higher than 0.50 for an input instance will classify it as ‘class A’; otherwise, ‘class B’. The LR model can be generalised to model a categorical variable with more than two values. This generalised version of LR is known as the multinomial logistic regression.

#### Support vector machine

**Fig. 2:**
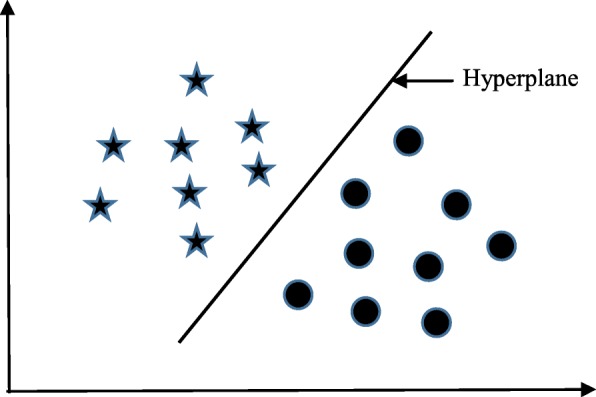
A simplified illustration of how the support vector machine works. The SVM has identified a hyperplane (actually a line) which maximises the separation between the ‘star’ and ‘circle’ classes

Support vector machine (SVM) algorithm can classify both linear and non-linear data. It first maps each data item into an n-dimensional feature space where *n* is the number of features. It then identifies the hyperplane that separates the data items into two classes while maximising the marginal distance for both classes and minimising the classification errors [23]. The marginal distance for a class is the distance between the decision hyperplane and its nearest instance which is a member of that class. More formally, each data point is plotted first as a point in an n-dimension space (where *n* is the number of features) with the value of each feature being the value of a specific coordinate. To perform the classification, we then need to find the hyperplane that differentiates the two classes by the maximum margin. Figure 2 provides a simplified illustration of an SVM classifier.

#### Decision tree

**Fig. 3:**
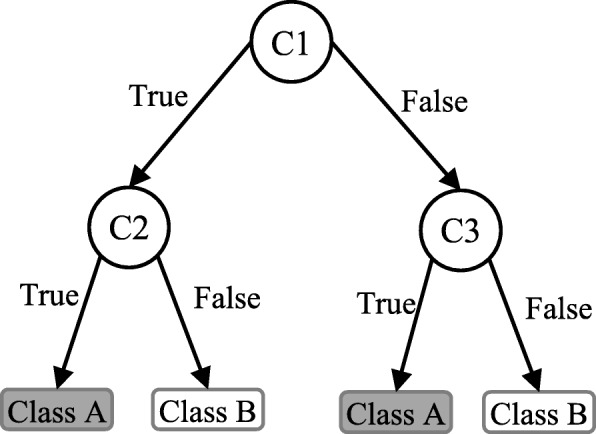
An illustration of a Decision tree. Each variable (C1, C2, and C3) is represented by a circle and the decision outcomes (Class A and Class B) are shown by rectangles. In order to successfully classify a sample to a class, each branch is labelled with either ‘True’ or ‘False’ based on the outcome value from the test of its ancestor node

Decision tree (DT) is one of the earliest and prominent machine learning algorithms. A decision tree models the decision logics i.e., tests and corresponds outcomes for classifying data items into a tree-like structure. The nodes of a DT tree normally have multiple levels where the first or top-most node is called the root node. All internal nodes (i.e., nodes having at least one child) represent tests on input variables or attributes. Depending on the test outcome, the classification algorithm branches towards the appropriate child node where the process of test and branching repeats until it reaches the leaf node [24]. The leaf or terminal nodes correspond to the decision outcomes. DTs have been found easy to interpret and quick to learn, and are a common component to many medical diagnostic protocols [25]. When traversing the tree for the classification of a sample, the outcomes of all tests at each node along the path will provide sufficient information to conjecture about its class. An illustration of an DT with its elements and rules is depicted in Fig. 3.

#### Random forest

**Fig. 4:**
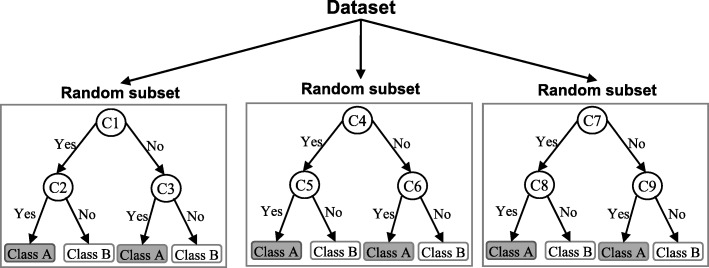
An illustration of a Random forest which consists of three different decision trees. Each of those three decision trees was trained using a random subset of the training data

A random forest (RF) is an ensemble classifier and consisting of many DTs similar to the way a forest is a collection of many trees [26]. DTs that are grown very deep often cause overfitting of the training data, resulting a high variation in classification outcome for a small change in the input data. They are very sensitive to their training data, which makes them error-prone to the test dataset. The different DTs of an RF are trained using the different parts of the training dataset. To classify a new sample, the input vector of that sample is required to pass down with each DT of the forest. Each DT then considers a different part of that input vector and gives a classification outcome. The forest then chooses the classification of having the most ‘votes’ (for discrete classification outcome) or the average of all trees in the forest (for numeric classification outcome). Since the RF algorithm considers the outcomes from many different DTs, it can reduce the variance resulted from the consideration of a single DT for the same dataset. Figure 4 shows an illustration of the RF algorithm.

#### Naïve Bayes

**Fig. 5:**
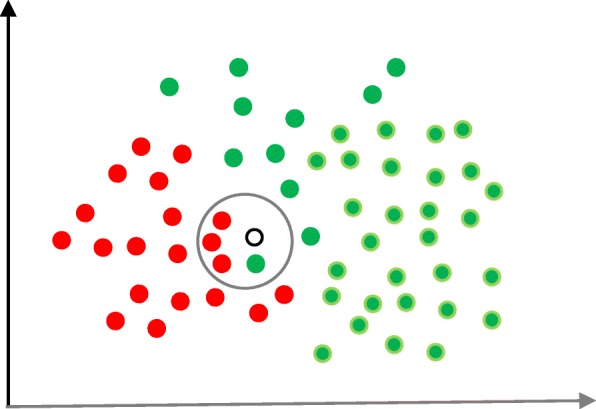
An illustration of the Naïve Bayes algorithm. The ‘white’ circle is the new sample instance which needs to be classified either to ‘red’ class or ‘green’ class

Naïve Bayes (NB) is a classification technique based on the Bayes’ theorem [27]. This theorem can describe the probability of an event based on the prior knowledge of conditions related to that event. This classifier assumes that a particular feature in a class is not directly related to any other feature although features for that class could have interdependence among themselves [28]. By considering the task of classifying a new object (white circle) to either ‘green’ class or ‘red’ class, Fig. 5 provides an illustration about how the NB technique works. According to this figure, it is reasonable to believe that any new object is twice as likely to have ‘green’ membership rather than ‘red’ since there are twice as many ‘green’ objects (40) as ‘red’. In the Bayesian analysis, this belief is known as the prior probability. Therefore, the prior probabilities of ‘green’ and ‘red’ are 0.67 (40 ÷ 60) and 0.33 (20 ÷ 60), respectively. Now to classify the ‘white’ object, we need to draw a circle around this object which encompasses several points (to be chosen prior) irrespective of their class labels. Four points (three ‘red’ and one ‘green) were considered in this figure. Thus, the likelihood of ‘white’ given ‘green’ is 0.025 (1 ÷ 40) and the likelihood of ‘white’ given ‘red’ is 0.15 (3 ÷ 20). Although the prior probability indicates that the new ‘white’ object is more likely to have ‘green’ membership, the likelihood shows that it is more likely to be in the ‘red’ class. In the Bayesian analysis, the final classifier is produced by combining both sources of information (i.e., prior probability and likelihood value). The ‘multiplication’ function is used to combine these two types of information and the product is called the ‘posterior’ probability. Finally, the posterior probability of ‘white’ being ‘green’ is 0.017 (0.67 × 0.025) and the posterior probability of ‘white’ being ‘red’ is 0.049 (0.33 × 0.15). Thus, the new ‘white’ object should be classified as a member of the ‘red’ class according to the NB technique.

#### K-nearest neighbour

**Fig. 6:**
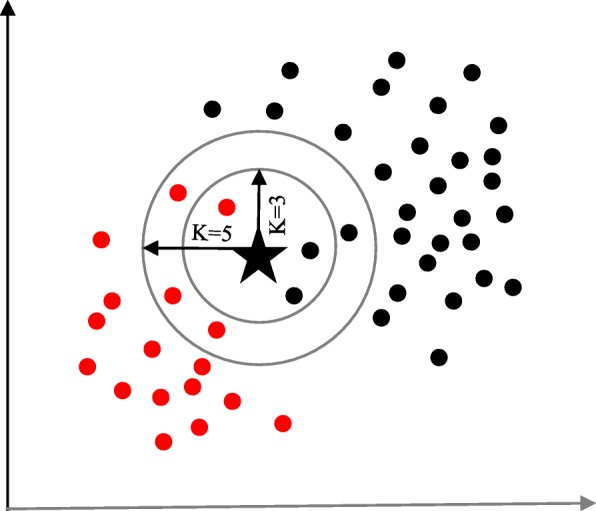
A simplified illustration of the K-nearest neighbour algorithm. When K = 3, the sample object (‘star’) is classified as ‘black’ since it gets more ‘vote’ from the ‘black’ class. However, for K = 5 the same sample object is classified as ‘red’ since it now gets more ‘vote’ from the ‘red’ class

The K-nearest neighbour (KNN) algorithm is one of the simplest and earliest classification algorithms [29]. It can be thought a simpler version of an NB classifier. Unlike the NB technique, the KNN algorithm does not require to consider probability values. The ‘*K*’ is the KNN algorithm is the number of nearest neighbours considered to take ‘vote’ from. The selection of different values for ‘*K*’ can generate different classification results for the same sample object. Figure 6 shows an illustration of how the KNN works to classify a new object. For *K = 3*, the new object (star) is classified as ‘black’; however, it has been classified as ‘red’ when *K = 5*.

#### Artificial neural network

**Fig. 7:**
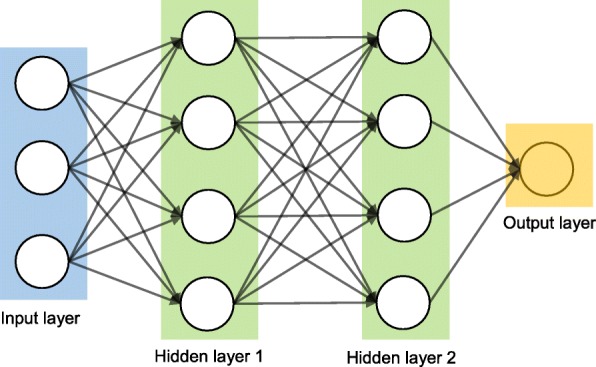
An illustration of the artificial neural network structure with two hidden layers. The arrows connect the output of nodes from one layer to the input of nodes of another layer

Artificial neural networks (ANNs) are a set of machine learning algorithms which are inspired by the functioning of the neural networks of human brain. They were first proposed by McCulloch and Pitts [30] and later popularised by the works of Rumelhart et al. in the 1980s [31].. In the biological brain, neurons are connected to each other through multiple axon junctions forming a graph like architecture. These interconnections can be rewired (e.g., through neuroplasticity) that helps to adapt, process and store information. Likewise, ANN algorithms can be represented as an interconnected group of nodes. The output of one node goes as input to another node for subsequent processing according to the interconnection. Nodes are normally grouped into a matrix called layer depending on the transformation they perform. Apart from the input and output layer, there can be one or more hidden layers in an ANN framework. Nodes and edges have weights that enable to adjust signal strengths of communication which can be amplified or weakened through repeated training. Based on the training and subsequent adaption of the matrices, node and edge weights, ANNs can make a prediction for the test data. Figure 7 shows an illustration of an ANN (with two hidden layers) with its interconnected group of nodes.

### Data source and data extraction

Extensive research efforts were made to identify articles employing more than one supervised machine learning algorithm for disease prediction. Two databases were searched (October 2018): Scopus and PubMed. Scopus is an online bibliometric database developed by Elsevier. It has been chosen because of its high level of accuracy and consistency [32]. PubMed is a free publication search engine and incorporates citation information mostly for biomedical and life science literature. It comprises more than 28 million citations from MEDLINE, life science journals and online books [33]. MEDLINE is a bibliographic database that includes bibliographic information for articles from academic journals covering medicine, nursing, pharmacy, dentistry, veterinary medicine, and health care [33].

A comprehensive search strategy was followed to find out all related articles. The search terms that were used in this search strategy were:

- “disease prediction” AND “machine learning”;
- “disease prediction” AND “data mining”;
- “disease risk prediction” AND “machine learning”; and
- “disease risk prediction” AND “data mining”.

In scientific literature, the generic name of “machine learning” is often used for both “supervised” and “unsupervised” machine learning algorithms. On the other side, there is a close relationship between the terms “machine learning” and “data mining”, with the latter is commonly used for the former one [34]. For these reasons, we used both “machine learning” and “data mining” in the search terms although the focus of this study is on the supervised machine learning algorithm. The four search items were then considered to launch searches on the titles, abstracts and keywords of an article for both Scopus and PubMed. This resulted in 305 and 83 articles from Scopus and PubMed, respectively. After combining these two lists of articles and removing the articles written in languages other than English, we found 336 unique articles.

Since the aim of this study was to compare the performance of different supervised machine learning algorithms, the next step was to select the articles from these 336 which used more than one supervised machine learning algorithm for disease prediction. For this reason, we wrote a computer program using Python programming language [35] which checked the presence of the name of more than one supervised machine learning algorithm in the title, abstract and keyword list of each of 336 articles. It found 55 articles that used more than one supervised machine learning algorithm for the prediction of different diseases. Out of the remaining 281 articles, only 155 used one of the seven supervised machine learning algorithms considered in this study. The rest 126 used either other machine learning algorithms (e.g., unsupervised or semi-supervised) or data mining methods other than machine learning ones. ANN was found most frequently (30.32%) in the 155 articles, followed by the Naïve Bayes (19.35%).

**Fig. 8:**
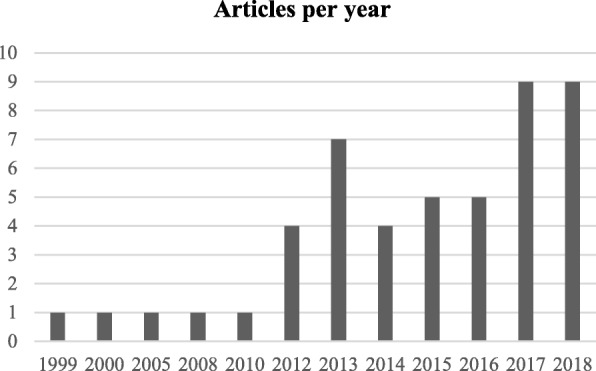
Number of articles published in different years

**Fig. 9:**
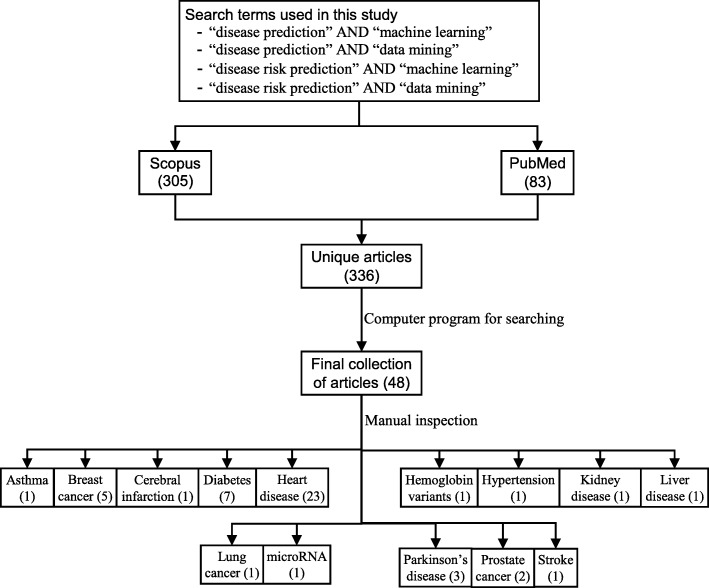
The overall data collection procedure. It also shows the number of articles considered for each disease

The next step is the manual inspection of all recovered articles. We noticed that four groups of authors reported their study results in two publication outlets (i.e., book chapter, conference and journal) using the same or different titles. For these four publications, we considered the most recent one. We further excluded another three articles since the reported prediction accuracies for all supervised machine learning algorithms used in those articles are the same. For each of the remaining 48 articles, the performance outcomes of the supervised machine learning algorithms that were used for disease prediction were gathered. Two diseases were predicted in one article [17] and two algorithms were found showing the best accuracy outcomes for a disease in one article [36]. In that article, five different algorithms were used for prediction analysis. The number of publications per year has been depicted in Fig. 8. The overall data collection procedure along with the number of articles selected for different diseases has been shown in Fig. 9.

**Fig. 10:**
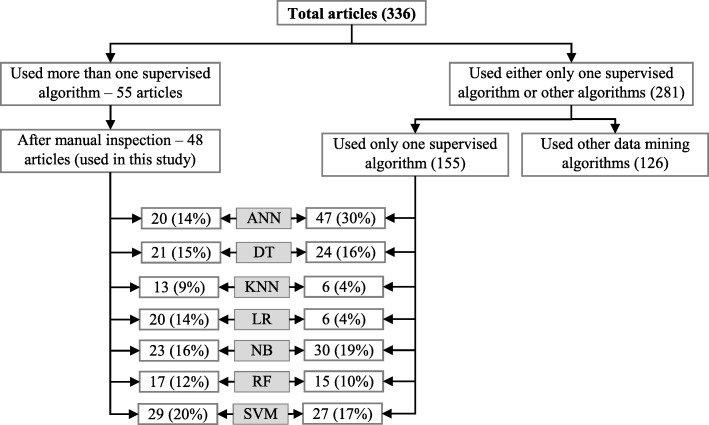
Composition of initially selected 329 articles with respect to the seven supervised learning algorithms

Figure 10 shows a comparison of the composition of initially selected 329 articles regarding the seven supervised machine learning algorithms considered in this study. ANN shows the highest percentage difference (i.e., 16%) between the 48 selected articles of this study and initially selected 155 articles that used only one supervised machine learning algorithm for disease prediction, which is followed by LR. The remaining five supervised machine learning algorithms show a percentage difference between 1 and 5.

### Classifier performance index

**Fig. 11:**
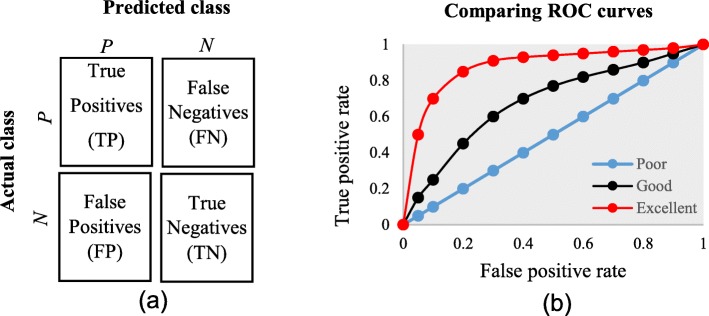
**a** The basic framework of the confusion matrix; and (**b**) A presentation of the ROC curve

The diagnostic ability of classifiers has usually been determined by the confusion matrix and the receiver operating characteristic (ROC) curve [37]. In the machine learning research domain, the confusion matrix is also known as error or contingency matrix. The basic framework of the confusion matrix has been provided in Fig. 11a. In this framework, true positives (TP) are the positive cases where the classifier correctly identified them. Similarly, true negatives (TN) are the negative cases where the classifier correctly identified them. False positives (FP) are the negative cases where the classifier incorrectly identified them as positive and the false negatives (FN) are the positive cases where the classifier incorrectly identified them as negative. The following measures, which are based on the confusion matrix, are commonly used to analyse the performance of classifiers, including those that are based on supervised machine learning algorithms.

$$ Accuracy=\frac{TP+ TN}{TP+ TN+ FP+ FN\ }\kern2em {F}_1\  score=\frac{2\times TP}{2\times TP+ FN+ FP} $$

$$ Precisioin=\frac{TP}{TP+ FP}\kern7.5em Sensitivity= Recall= True\ positive\ rate=\frac{TP}{TP+ FN} $$

$$ Specificity=\frac{TN}{TN+ FP\ }\kern6.75em False\ positive\ rate=\frac{FP}{FP+ TN\ } $$

An ROC is one of the fundamental tools for diagnostic test evaluation and is created by plotting the true positive rate against the false positive rate at various threshold settings [37]. The area under the ROC curve (AUC) is also commonly used to determine the predictability of a classifier. A higher AUC value represents the superiority of a classifier and vice versa. Figure 11b illustrates a presentation of three ROC curves based on an abstract dataset. The area under the blue ROC curve is half of the shaded rectangle. Thus, the AUC value for this blue ROC curve is 0.5. Due to the coverage of a larger area, the AUC value for the red ROC curve is higher than that of the black ROC curve. Hence, the classifier that produced the red ROC curve shows higher predictive accuracy compared with the other two classifiers that generated the blue and red ROC curves.

There are few other measures that are also used to assess the performance of different classifiers. One such measure is the running mean square error (RMSE). For different pairs of actual and predicted values, RMSE represents the mean value of all square errors. An error is the difference between an actual and its corresponding predicted value. Another such measure is the mean absolute error (MAE). For an actual and its predicted value, MAE indicates the absolute value of their difference.

## Results

The final dataset contained 48 articles, each of which implemented more than one variant of supervised machine learning algorithms for a single disease prediction. All implemented variants were already discussed in the methods section as well as the more frequently used performance measures. Based on these, we reviewed the finally selected 48 articles in terms of the methods used, performance measures as well as the disease they targeted.

In Table 1, names and references of the diseases and the corresponding supervised machine learning algorithms used to predict them are discussed. For each of the disease models, the better performing algorithm is also described in this table. This study considered 48 articles, which in total made the prediction for 49 diseases or conditions (one article predicted two diseases [17]). For these 49 diseases, 50 algorithms were found to show the superior accuracy. One disease has two algorithms (out of 5) that showed the same higher-level accuracies [36]. To sum up, 49 diseases were predicted in 48 articles considered in this study and 50 supervised machine learning algorithms were found to show the superior accuracy. The advantages and limitations of different supervised machine learning algorithms are shown in Table 2.

**Table 1:** Summary of all references

Reference	Disease predicted	Algorithms compared	Type of data	Number of subjects	Cross validation method	Prediction performance	Best one (s)
Aneja and Lal [38]	Asthma	ANN, NB	Disease symptom	1024	–	Accuracy (ANN = 85, NB = 88)	NB
Ayer et al. [39]	Breast cancer	ANN, LR	Clinical and demographic data	62,219	10-fold cross validation	AUC (ANN = 0.965, LR = 0.963)	ANN
Ahmad et al. [18]	Breast cancer	ANN, DT, SVM	Clinical data for cancer incidence and survival	1189	10-fold cross validation	Accuracy (ANN = 0.947, DT = 0.936, SVM = 0.957) Sensitivity (ANN = 0.956, DT = 0.958, SVM = 0.971) Specificity (ANN = 0.928, DT = 0.907, SVM = 0.945)	SVM
Lundin et al. [40]	Breast cancer	ANN, LR	Clinical and demographic data	951	–	AUC (ANN = 0.909, LR = 0.897)	ANN
Delen et al. [41]	Breast cancer	ANN, DT, LR	Clinical and demographic data	202,932	10-fold cross validation	Accuracy (ANN = 0.909, DT = 0.935, LR = 0.894)	DT
Yao et al. [8]	Breast cancer	DT, RF, SVM	Image data	569	10-fold cross validation	Accuracy (DT = 0.932, RF = 0.963, SVM = 0.959)	RF
Chen et al. [42]	Cerebral infarction	DT, KNN, NB	Electronic health records, medical image and gene data	31,919	10-fold cross validation	AUC (DT = 0.646, KNN = 0.454, NB = 0.495)	DT
Cai et al. [43]	Diabetes	LR, NB, SVM	Gut microbiota	489	10-fold cross validation	AUC (LR = 0.98, NB = 0.94, SVM = 0.99)	SVM
Malik et al. [44]	Diabetes	ANN, LR, SVM	Electrochemical measurements of saliva	175	3-fold cross validation	Accuracy (ANN = 80.70, LR = 75.86, SVM = 84.09) F_1_ score (ANN = 80.20, LR = 75.71, SVM = 84.06)	SVM
Farran [17]	Diabetes	KNN, LR, SVM	Demographic, anthropometric, vital signs, diagnostic and clinical lab measurement data	10,632	5-fold cross validation	Accuracy (KNN = 79.5, LR = 80.7, SVM = 82.6)	SVM
Mani et al. [45]	Diabetes	KNN, LR, NB, RF, SVM	Demographic and clinical test result	2280	5-fold cross validation	AUC (KNN = 0.721, LR = 0.755, NB = 0.762, RF = 0.803, SVM = 0.749)	RF
Tapak et al. [46]	Diabetes	ANN, LR, RF, SVM	Demographic, anthropometric, diagnostic and clinical lab measurement data	6500	10-fold cross validation	Accuracy (ANN = 0.931, LR = 0.935, RF = 0.930, SVM = 0.986) AUC (ANN = 0.751, LR = 0.763, RF = 0.717, SVM = 0.979)	SVM
Sisodia and Sisodia [47]	Diabetes	DT, NB, SVM	Clinical test result	768	10-fold cross validation	Accuracy (DT = 0.738, NB = 0.763, SVM = 0.651)	NB
Yang et al. [48]	Diabetes	RF, SVM	Clinical and gene expression data	9343	10-fold cross validation	Accuracy (RF = 0.742, SVM = 0.723)	RF
Juhola et al. [49]	Heart disease	KNN, RF, SVM	Signal data	–	–	Accuracy (84.5, RF = 87.6, SVM = 87.1)	RF
Long et al. [50]	Heart disease	ANN, NB, SVM	Clinical, demographic and image data	537	–	Accuracy (ANN = 77.8, NB = 83.3, SVM = 75.9	NB
Palaniappan and Awang [21]	Heart disease	ANN, DT, NB	Clinical and demographic data	909	2-fold cross validation	Accuracy (ANN = 85.682, DT = 78.8334, NB = 87.885)	NB
Jin et al. [51]	Heart disease	LR, RF	Electronic health records	20,000	5-fold cross validation	AUC (LR = 0.663, RF = 0.627)	LR
Puyalnithi and Viswanatham [52]	Heart disease	DT, NB, RF, SVM	Clinical and demographic data	746	k-fold and leave-one-out	AUC (DT = 0.940, NB = 0.942, RF = 0.917, SVM = 0.731)	NB
Forssen et al. [53]	Heart disease	LR, RF	Metabolomic data	3409	50-fold cross validation	Accuracy (LR = 0.767, RF = 0.732) AUC (LR = 0.765, RF = 0.711)	LR
Tang et al. [54]	Heart disease	ANN, LR	Clinical, demographic, behavioural and medical data	2092	–	AUC (ANN = 0.762, LR = 0.758) Accuracy (ANN = 0.714, LR = 0.698)	ANN
Toshniwal et al. [55]	Heart disease	NB, RF, SVM	Electrocardiography data	47	–	Accuracy (NB = 88.44, RF = 98.49, SVM = 98.41)	RF
Alonso et al. [56]	Heart disease	LR, SVM	Clinical data	8321	5-fold cross validation	AUC (LR = 0.76 and SVM = 0.83)	SVM
Mustaqeem et al. [57]	Heart disease	KNN, NB, RF, SVM	Electrocardiography data	452	10-fold cross validation	Accuracy (KNN = 76.60, NB = 74.43, RF = 76.50, SVM = 74.47)	KNN
Mansoor et al. [58]	Heart disease	LR, RF	Demographic and hospital admission	9637	10-fold cross validation	Accuracy (LR = 0.88, RF = 0.89)	RF
Kim et al. [59]	Heart disease	ANN, DT, LR, SVM	Demographic, behavioural and disease data	748	–	AUC (ANN = 0.663, DT = 0.631, LR = 0.658, SVM = 0.664)	SVM
Kim et al. [59]	Heart disease	ANN, LR	Demographic, behavioural and disease data	4146	–	Accuracy (ANN = 87.04, LR = 86.11)	ANN
Taslimitehrani et al. [60]	Heart disease	DT, LR, RF, SVM	Electronic health records	119,749	2-fold cross validation	AUC (DT = 0.66, LR = 0.81, RF = 0.80, SVM = 0.59)	LR
Anbarasi et al. [61]	Heart disease	DT, NB	Clinical and demographic data	909	k-fold cross validation	Accuracy (DT = 99.2%, NB = 96.5%)	DT
Bhatla and Jyoti [62]	Heart disease	ANN, DT, NB	Clinical data	3000	10-fold cross validation	Accuracy (ANN = 85.53%, DT = 89%, NB = 86.53%)	DT
Thenmozhi and Deepika [63]	Heart disease	ANN, DT, NB	Clinical data and medical diagnostic data	–	10-fold cross validation	Accuracy (ANN = 99.25, DT = 96.66, NB = 94.44)	ANN
Tamilarasi and Porkodi [64]	Heart disease	ANN, KNN, NB	Clinical and demographic data	–	–	Accuracy (ANN = 99.25, KNN = 100, NB = 85.92)	KNN
Marikani and Shyamala [65]	Heart disease	DT, KNN, NB, RF, SVM	Clinical and demographic data	303	–	Accuracy (DT = 0.954, KNN = 0.757, NB = 0.817, RF = 0.963, SVM = 1.0)	SVM
Lu et al. [66]	Heart disease	ANN, NB, SVM	Clinical, demographic and diagnostic data	1090	–	Accuracy (ANN = 86.04, NB = 82.31, SVM = 86.62)	SVM
Khateeb and Usman [67]	Heart disease	DT, KNN, NB	Clinical and demographic data	303	10-fold cross validation	Accuracy (DT = 76.89, KNN = 79.20, NB = 66.66)	KNN
Patel et al. [68]	Heart disease	DT, NB	Clinical and demographic data	–	–	Accuracy (DT = 99.2, NB = 96.5)	DT
Venkatalakshmi and Shivsankar [69]	Heart disease	DT, NB	Clinical and demographic data	294	–	Accuracy (DT = 84.01, NB = 85.03)	DT
Borah et al. [36]	Hemoglobin variants	DT, KNN, LR, RF, SVM	Clinical and demographic data	1500	–	DT and RF (Precision = 93.84, Recall = 92.78, F_1_ score = 93.33) Precision (KNN = 92.23, LR = 89.23, SVM = 66.67) Recall (KNN = 91.67, LR = 87.34, SVM = 64.78) F_1_ score (KNN = 91.95, LR = 88.27, SVM = 65.71)	DT, RF
Farran [17]	Hypertension	KNN, LR, SVM	Demographic, anthropometric, vital signs, diagnostic and clinical lab measurement data	10,632	5-fold cross validation	Accuracy (KNN = 82.4, LR = 82.1, SVM = 83)	SVM
Ani et al. [70]	Kidney disease	ANN, DT, KNN, NB	Clinical and demographic data	400	10-fold cross validation	Accuracy (ANN = 81, DT = 93, KNN = 90, NB = 78)	DT
Islam et al. [71]	Liver disease	ANN, LR, RF, SVM	Clinical, demographic and ultrasonography test data	994	10-fold cross validation	Accuracy (ANN = 0.691, LR = 0.707, RF = 0.658, SVM = 0.690) AUC (ANN = 0.733, LR = 0.763, RF = 0.708, SVM = 0.657)	LR
Lynch et al. [72]	Lung cancer	DT, RF, SVM	Clinical and demographic data	–	10-fold cross validation	Running Mean Square Error (DT = 15.81, RF = 15.63, SVM = 15.82)	RF
Chen et al. [73]	microRNA	RF, SVM	microRNA data	96,325	5-fold cross validation	Accuracy (RF = 75.24, SVM = 70.02)	RF
Eskidere et al. [74]	Parkinson’s disease	ANN, SVM	Voice recording and demographic data	42	10-fold cross validation	Mean absolute error (SVM = 6.99), ANN = 8.20)	SVM
Chen et al. [75]	Parkinson’s disease	KNN, SVM	Voice recording and demographic data	31	10-fold cross validation	Accuracy (KNN = 95.78, SVM = 93.52) AUC (KNN = 95.60, SVM = 91.12)	KNN
Behroozi and Sami [76]	Parkinson’s disease	KNN, NB, SVM	Voice recording and demographic data	40	Leave-one-out	Accuracy (KNN = 77.50, NB = 80.00, SVM = 87.50)	SVM
Hussain et al. [77]	Prostate cancer	DT, NB, SVM	Magnetic resonance imaging data	20	10-fold cross validation	AUC (DT = 0.955, NB = 0.989, SVM = 0.997)	SVM
Zupan et al. [78]	Prostate cancer	DT, NB	Clinical data	2051	10-fold cross validation	Accuracy (NB = 70.80, DT = 68.80)	NB
Hung et al. [79]	Stroke	ANN, LR, SVM	Electronic medical claim and demographic data	798,611	–	Accuracy (ANN = 0.873, LR = 0.866, SVM = 0.839)	ANN

**Table 2:** Advantages and limitations of different supervised machine learning algorithms

Supervised algorithm	Advantages	Limitations
Artificial neural network (ANN)	- Can detect complex nonlinear relationships between dependent and independent variables. - Requires less formal statistical training. - Availability of multiple training algorithms. - Can be applied to both classification and regression problems.	- Have characteristics of ‘black box’ - user can not have access to the exact decision-making process and therefore, - Computationally expensive to train the network for a complex classification problem. - Predictor or Independent variables require pre-processing.
Decision tree (DT)	- Resultant classification tree is easier to understand and interpret. - Data preparation is easier. - Multiple data types such as numeric, nominal, categorical are supported. - Can generate robust classifiers and can be validated using statistical tests.	- Require classes to be mutually exclusive. - Algorithm cannot branch if any attribute or variable value for a non-leaf node is missing. - Algorithm depends on the order of the attributes or variables. - Do not perform as well as some other classifier (e.g., Artificial Neural Network) [80]
K-nearest neighbour (KNN)	- Simple algorithm and can classify instances quickly. - Can handle noisy instances or instances with missing attribute values. - Can be used for classification and regression.	- Computationally expensive as the number of attributes increases. - Attributes are given equal importance, which can lead to poor classification performance. - Provide no information on which attributes are most effective in making a good classification.
Logistic regression (LR)	- Easy to implement and straightforward. - LR-based models can be updated easily. - Does not make any assumptions regarding the distribution of independent variable (s). - It has a nice probabilistic interpretation of model parameters.	- Does not have good accuracy when input variables have complex relationships. - Does not consider the linear relationship between variables. - Key components of LR - logic models, are vulnerable to overconfidence. - May overstate the prediction accuracy due to sampling bias. - Unless multinomial, generic LR can only classify variables that have two states (i.e., dichotomous).
Naïve Bayes (NB)	- Simple and very useful for large datasets. - Can be used for both binary and multi-class classification problems. - It requires less amount of training data. - It can make probabilistic predictions and can handle both continuous and discrete data.	- Classes must be mutually exclusive. - Presence of dependency between attributes negatively affects the classification performance. - It assumes the normal distribution of numeric attributes.
Random forest (RF)	- Lower chance of variance and overfitting of training data compared to DT, since RF takes the average value from the outcomes of its constituent decision trees. - Empirically, this ensemble-based classifier performs better than its individual base classifiers, i.e., DTs. - Scales well for large datasets. - It can provide estimates of what variables or attributes are important in the classification.	- More complex and computationally expensive. - Number of base classifiers needs to be defined. - It favours those variables or attributes that can take high number of different values in estimating variable importance. - Overfitting can occur easily.
Support vector machine (SVM)	- More robust compared to LR - Can handle multiple feature spaces. - Less risk of overfitting. - Performs well in classifying semi-structured or unstructured data, such as texts, images etc.	- Computationally expensive for large and complex datasets. - Does not perform well if the data have noise. - The resultant model, weight and impact of variables are often difficult to understand. - Generic SVM cannot classify more than two classes unless extended.

The comparison of the usage frequency and accuracy of different supervised learning algorithms are shown in Table 3. It is observed that SVM has been used most frequently (29 out of 49 diseases that were predicted). This is followed by NB, which has been used in 23 articles. Although RF has been considered the second least number of times, it showed the highest percentage (i.e., 53%) in revealing the superior accuracy followed by SVM (i.e., 41%).

**Table 3:** Comparison of usage frequency and accuracy of different supervised machine learning algorithms

Supervised machine learning algorithms	Number of published articles used this algorithm	Number of times this algorithm showed superior accuracy (%)
Artificial neural network (ANN)	20	6 (30%)
Decision tree (DT)	21	7 (33%)
K-nearest neighbour (KNN)	13	4 (31%)
Logistic regression (LR)	20	5 (25%)
Naïve Bayes (NB)	23	7 (30%)
Random forest (RF)	17	9 (53%)
Support vector machine (SVM)	29	13 (41%)

In Table 4, the performance comparison of different supervised machine learning algorithms for most frequently modelled diseases is shown. It is observed that SVM showed the superior accuracy at most times for three diseases (e.g., heart disease, diabetes and Parkinson’s disease). For breast cancer, ANN showed the superior accuracy at most times.

**Table 4:** Comparison of the performance of different supervised machine learning algorithms based on different criteria

Criteria	# articles meet this criterion (%)	Name and frequency of the algorithm that showed ‘superior’ accuracy	
		*Most times*	*Second most times*
Disease names that were frequently modelled			
Heart disease	23 (48%)	NB, SVM (4 times, each)	ANN, DT, KNN, LR (3 times, each)
Diabetes	7 (15%)	SVM (4 times)	RF (2 times)
Breast cancer	5 (10%)	ANN (2 times)	DT, RF, SVM (1 time, each)
Parkinson’s disease	3 (6%)	SVM (2 times)	KNN (1 time)
Type of the data that were used			
Clinical and Demographical	15 (30%)	DT (6)	ANN, KNN, NB, RF (2 times, each)
Other data types	33 (66%)	SVM, RF (12 times, each)	RF (7)
Validation method followed			
10-fold validation	21 (42%)	SVM (5 times)	DT, RF (4 times, each)
5-fold validation	6 (12%)	SVM (3 times)	RD (2 times)
Other method	7 (14%)	LR, NB, SVM (2 times, each)	DT (1 time)
Do not use any method	16 (32%)	ANN (4 times)	DT, RF, SVM (3 times, each)

**Fig. 12:**
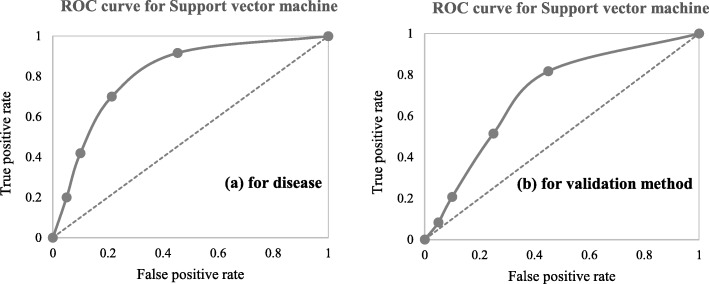
Illustration of the superior performance of the Support vector machine using ROC graphs (based on the data from Table 4) – (**a**) for disease names that were modelled; and (**b**) for validation methods that were followed

A close investigation of Table 1 reveals an interesting result regarding the performance of different supervised learning algorithms. This result has also been reported in Table 4. Consideration of only those articles that used clinical and demographic data (15 articles) reveals DT as to show the superior result at most times (6). Interestingly, SVM has been found the least time (1) to show the superior result although it showed the superior accuracy at most times for heart disease, diabetes and Parkinson’s disease (Table 4). In other 33 articles that used research data other than ‘clinical and demographic’ type, SVM and RF have been found to show the superior accuracy at most times (12) and second most times (7), respectively. In articles where 10-fold and 5-fold validation methods were used, SVM has been found to show the superior accuracy at most times (5 and 3 times, respectively). On the other side, articles where no method was used for validation, ANN has been found at most times to show the superior accuracy. Figure 12 further illustrates the superior performance of SVM. Performance statistics from Table 4 have been used in a normalised way to draw these two graphs. Fig. 12a illustrates the ROC graph for the four diseases (i.e., Heart disease, Diabetes, Breast cancer and Parkinson’s disease) under the ‘*disease names that were modelled*’ criterion. The ROC graph based on the ‘*validation method followed*’ criterion has been presented in Fig. 12b.

## Discussion

To avoid the risk of selection bias, from the literature we extracted those articles that used more than one supervised machine learning algorithm. The same supervised learning algorithm can generate different results across various study settings. There is a chance that a performance comparison between two supervised learning algorithms can generate imprecise results if they were employed in different studies separately. On the other side, the results of this study could suffer a variable selection bias from individual articles considered in this study. These articles used different variables or measures for disease prediction. We noticed that the authors of these articles did not consider all available variables from the corresponding research datasets. The inclusion of a new variable could improve the accuracy of an underperformed algorithm considered in the underlying study, and vice versa. This is one of the limitations of this study. Another limitation of this study is that we considered a broader level classification of supervised machine learning algorithms to make a comparison among them for disease prediction. We did not consider any sub-classifications or variants of any of the algorithms considered in this study. For example, we did not make any performance comparison between least-square and sparse SVMs; instead of considering them under the SVM algorithm. A third limitation of this study is that we did not consider the hyperparameters that were chosen in different articles of this study in comparing multiple supervised machine learning algorithms. It has been argued that the same machine learning algorithm can generate different accuracy results for the same data set with the selection of different values for the underlying hyperparameters [81, 82]. The selection of different kernels for support vector machines can result a variation in accuracy outcomes for the same data set. Similarly, a random forest could generate different results, while splitting a node, with the changes in the number of decision trees within the underlying forest.

## Conclusion

This research attempted to study comparative performances of different supervised machine learning algorithms in disease prediction. Since clinical data and research scope varies widely between disease prediction studies, a comparison was only possible when a common benchmark on the dataset and scope is established. Therefore, we only chose studies that implemented multiple machine learning methods on the same data and disease prediction for comparison. Regardless of the variations on frequency and performances, the results show the potential of these families of algorithms in the disease prediction.

## Data Availability

The data used in this study can be extracted from online databases. The detail of this extraction has been described within the manuscript.

## Abbreviations

ANN: Artificial neural network
AUC: Area under the ROC curve
DT: Decision Tree
FN: False negative
FP: False positive
KNN: K-nearest neighbour
LR: Logistic regression
MAE: Mean absolute error
NB: Naïve Bayes
RF: Random forest
RMSE: Running mean square error
ROC: Receiver operating characteristic
SVM: Support vector machine
TN: True negative
TP: True positive
